# Ferric Chloride Complexes in Aqueous Solution: An EXAFS Study

**DOI:** 10.1007/s10953-018-0756-6

**Published:** 2018-05-05

**Authors:** Ingmar Persson

**Affiliations:** Department of Molecular Sciences, Swedish University of Agricultural Sciences, P.O. Box 7015, 750 07 Uppsala, Sweden

**Keywords:** EXAFS, Ferric chloride, Aqueous solution, *trans*-[FeCl_2_(H_2_O)_4_]^+^ complex

## Abstract

A series of concentrated aqueous solutions of ferric chloride with different chloride:iron(III) ratios has been studied by means of EXAFS to determine the structure around the iron(III) ion of the dominating species in such solutions. The dominating species in dilute acidic aqueous solution of ferric chloride, at less than 1 mmol·dm^−3^, are the hydrated iron(III) and chloride ions, while in concentrated aqueous solution and in solutions with an excess of chloride ions, up to 1.0 mol·dm^−3^, it is the *trans*-[FeCl_2_(H_2_O)_4_]^+^ complex. Possible higher chloroferrate(III) or dimeric [Fe_2_Cl_6_] complexes at room temperature, as proposed in the literature, were not observed in any of the studied solutions in spite of an excess of chloride ions of 1 mol·dm^−3^.

**Electronic supplementary material:**

The online version of this article (10.1007/s10953-018-0756-6) contains supplementary material, which is available to authorized users.

## Introduction

Ferric chloride, or iron(III) chloride, is a very important chemical used in several branches of industry [1–4]. Iron(III) chloride is produced as an aqueous solution from the oxidation of ferrous chloride with chlorine. The primary use of ferric chloride is to remove impurities in water and for wastewater treatment. Including industrial water treatment applications and its use in pre-treatment of seawater prior to desalination, the total water treatment accounts for approximately 80% of the total demand of ferric chloride globally [1]. The second-largest application is in the production of printed circuit boards, which accounts for ca. 10% of the demand [1]. Other uses of ferric chloride are as a leaching agent in chloride hydrometallurgy [2], for example in the production of silicon from FeSi (the Silgrain process) [3], and as catalyst for the reaction of ethylene with chlorine forming ethylene dichloride (1,2-dichloroethane) [4], an important commodity chemical mainly used for the industrial production of vinyl chloride. Ferric chloride has the unusual distinction of being one of the purest and most concentrated forms of iron commercially available. However, what is truly unusual is that ferric chloride not only functions as a reactant to remove water impurities but it also acts both as a coagulant and flocculant. Further popular information about ferric chloride can be found at the homepage of the chemical of the month [5].

Complex formation in the ferric chloride system has been studied for a century, showing weak complex formation in dilute aqueous solution, while the complex formation is significantly stronger in hyper-saline aqueous solutions and at elevated temperatures [6], and in aprotic solvents such as dimethyl sulfoxide and *N*,*N*-dimethylformamide. A selection of the reported stability constants in the ferric chloride system in aqueous and non-aqueous systems is given in supplementary Table S1.

A number of large angle X-ray scattering investigations were carried out many years ago on the ferric chloride system [7–16]. Acidified and neutral aqueous solutions of FeCl_3_·6H_2_O of various chemical compositions together with a FeCl_3_·6H_2_O melt have been investigated in order to reveal the structures of the solutes present in solution. The conclusions given by different authors are contradictory. The main disagreement concerns the chemical species dominating in concentrated aqueous solution. Magini et al. concluded that complex formation between Fe^3+^ and Cl^−^ ions occurs in freshly prepared solution, no matter the concentration, with octahedral chloro complexes [Fe(H_2_O)_6−*n*_Cl_*n*_]^3−^ (*n* = 1–3) or tetrahedral [FeCl_4_]^−^ ions as the main species in such solutions [11, 12, 16]. It has been claimed that the dimeric [Fe_2_Cl_6_] complex is present in 5 mol·dm^−3^ aqueous solutions of iron(III) chloride [7, 9], and in the FeCl_3_·6H_2_O melt [12]. The [Fe_2_Cl_6_] complex is composed of two tetrahedra sharing an edge [12]; the same species have also been suggested in the gaseous state by electron diffraction [17–19] and in methanol solution [20]. Wertz and co-workers reported that different species are acquired, even for solutions having the same concentration, if prepared from different starting materials, i.e. anhydrous ferric chloride and ferric chloride hexahydrate, respectively [13, 14]. They proposed that hydrated iron(III) ions, [Fe(H_2_O)_6_]^3+^, are present in a freshly prepared solution of FeCl_3_·6H_2_O, without any complex formation, but octahedral chloro complexes [FeCl_*x*_(H_2_O)_6−*x*_]^(3−*x*)+^ are gradually formed with an average composition [FeCl_1.4_(H_2_O)_4.6_]^1.6+^ after aging the solution for 14 months [14]. On the other hand, the tetrahedral tetrachloro complexes, [FeCl_4_]^−^, was proposed to be predominant in a solution freshly prepared from anhydrous FeCl_3_. Giubileo et al. [16] contradicted these claims, proving that the chemical species formed are independent of starting material and no aging effect was detected. Without providing any evidence, Wertz et al. [13], assumed that the dimer [Fe_2_Cl_6_] should exist in 4 mol·dm^−3^ aqueous solution prepared from anhydrous FeCl_3_. It was pointed out that species of the type [FeCl_5_(H_2_O)]^2−^ or [FeCl_6_]^3−^ are excluded due to their instability in solution even though they can be stabilized in the solid state, Table S2 [12, 21]. An EXAFS and UV–Vis spectrophotometric study of ferric chloride in hyper-saline LiCl aqueous solutions showed that tetrachloroferrate(III) dominates in solutions that are at least 10 mol·kg^−1^ in chloride, and the [FeCl_3_(H_2_O)_*n*_], *n* = 1 or 2, complexes at somewhat lower chloride concentrations [22]. That study also showed that the stability of higher iron(III)–chloride complexes increases with increasing temperature.

Solid FeCl_3_·6H_2_O consists of octahedral *trans*-[Fe(H_2_O)_4_Cl_2_]^+^ and chloride ions with Fe–Cl and Fe–O bond distances of 2.296 and 2.070 Å, respectively [23]. The octahedral *trans*-[Fe(H_2_O)_4_Cl_2_]^+^ unit is also present in solid *trans*-[FeCl_2_(H_2_O)_4_][SbCl_6_]·4H_2_O with reported Fe–Cl and Fe–O bond distances of 2.364 and 2.077 Å, respectively [24]. An octahedral *cis*-[Fe(H_2_O)_4_Cl_2_]^+^ complex has been reported in *cis*-[FeCl_2_(H_2_O)_4_][FeCl_4_]·H_2_O with Fe–Cl and Fe–O bond distances of 2.245 and 2.020 Å, respectively [25].

The literature survey of the speciation of the ferric chloride system in aqueous solution, summarized above, shows clearly a scattered picture of the speciation and the structure of the dominating species in this system. This is only the second EXAFS (extended X-ray absorption fine structure) spectroscopy study applied on this system. EXAFS gives more accurate structural parameters than LAXS for short distances to the absorbing atom, in this case iron, than the previously applied large angle X-ray scattering method [26]. Furthermore, the structural data on the ferric chloride system aqueous solution were reported at least 35 years ago, and the accuracy of the LAXS and EXAFS techniques has improved significantly over this time. In the present work, Fe K-edge EXAFS spectra have been collected in order to improve the knowledge of the structure of the chemical species present in these aqueous solutions of iron(III) chloride with different concentrations and iron(III):chloride ratios, that are widely used industrially.

## Experimental Section

### Preparation of Samples

The aqueous solutions were prepared by dissolving the appropriate amounts of FeCl_3_·6H_2_O (Merck, AnalaR NORMAPUR ACS analytical reagent, min. 99%) in dilute hydrochloric acid and as a very concentrated aqueous solution to suppress the formation of hydrolysis products [27–30] and promote the formation of higher chloride complexes. The chemical composition of the studied solutions and their labelling are given in Table 1. 25.2 mg solid *trans*-[FeCl_2_(OH_2_)_4_]Cl·2H_2_O was mixed with 45 mg boron nitride (BN) and carefully ground and pressed into a 1.0 mm thick aluminum frame for the EXAFS study.

**Table 1:** Concentrations (mol·dm^−3^) of the aqueous iron(III) chloride solutions studied by EXAFS

Solution	[Fe^3+^]	[Cl^−^]	$[{\text{ClO}}_{4}^{ - } ]$	[H^+^]	$\bar{n}$
Cl1	1.00	1.50	1.60	0.10	1.10
Cl2	1.00	3.00	–	–	1.60
Cl3	1.00	3.00	1.00	1.00	1.60
Cl4	2.33	6.99	0.10	0.10	1.78
Cl5	1.00	4.00	–	1.00	1.73

The $\bar{n}$ values, i.e. the mean number of chloride ions per iron(III) ion, were calculated from the stability constants given by Strahm et al. [33] for the iron(III) chloride system in water, *I* = 2.6 mol·dm^−3^ NaClO_4_, *T *= 293 K, *K*_1_ = 6.46 mol^−1^·dm^3^ and *K*_2_ = 1.8 mol^−1^·dm^3^

### Exafs

Iron K edge X-ray absorption spectra of the mentioned samples were collected at the Stanford Synchrotron Radiation Lightsource (SSRL), beam line 4-1 (old station). Transmission and fluorescence data were collected simultaneously using ion chambers with a gentle nitrogen flow and a Lytle detector with krypton gas. The spectrum of an iron foil was recorded simultaneously in transmission mode for internal energy calibration with the first inflection point of metallic iron defined as 7111.3 keV [31]. Higher-order harmonics were reduced by detuning the second monochromator crystal to reflect 50% of the maximum intensity at the end of the scans. The aqueous solutions were contained in cells with 1.0 mm Teflon spacers and 6 μm polyethylene X-ray film windows hold together with titanium frames. The experiments were performed at room temperature using the synchrotron radiation provided from a 3 GeV storage ring and monochromatized by a Si[220] double crystal monochromator. In order to determine the solute structures of the aqueous iron(III) chloride solutions the EXAFS oscillations were extracted using standard procedures for pre-edge, subtraction, spline removal and data normalization. For each sample six scans were averaged. All data treatment was performed with use of the EXAFSPAK program package [32]. To obtain quantitative information the *k*^3^-weighted EXAFS oscillations were analyzed by nonlinear least-squares fitting of the model parameters. Model fitting was performed with theoretical phase and amplitude functions including both single and multiple scattering paths using the ab initio code FEFF7 (version 7.02) [33].

The standard deviations given for the reported refined parameters are obtained from *k*^3^-weighted least-squares refinements of the EXAFS function *χ*(*k*) and do not include systematic errors of the measurements. These statistical error estimates provide a measure of the precision of the results and allow reasonable comparisons, for example, of the significance of relative shifts in the distances. However, the variations in the refined parameters, including the shift in the *E*_0_ value (for which *k *= 0), for different models and data ranges, indicate that the absolute accuracy of the distances given for the separate complexes is within ± 0.005–0.02 Å for well-defined interactions. The “standard deviations” given in the text have been increased accordingly to include estimated additional effects of systematic errors.

## Results and Discussion

**Fig. 1:**
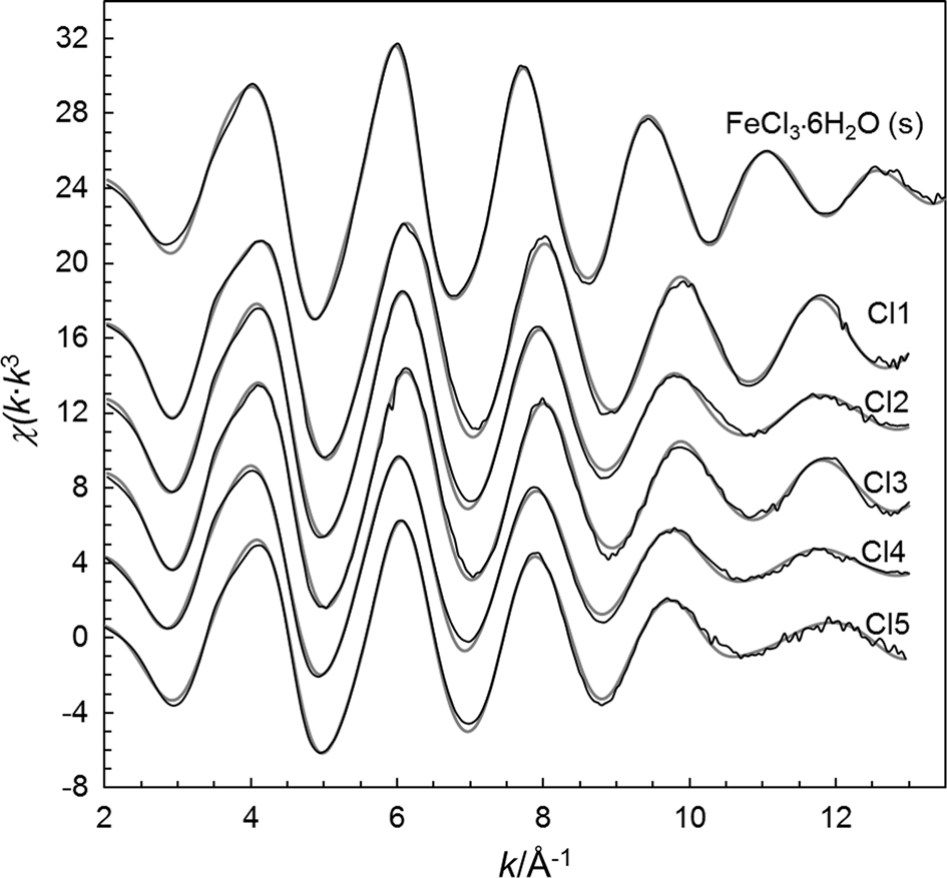
Fitting of the raw EXAFS data (black thin lines) using the structure parameters summarized in Table 2 (grey think lines)

**Fig. 2:**
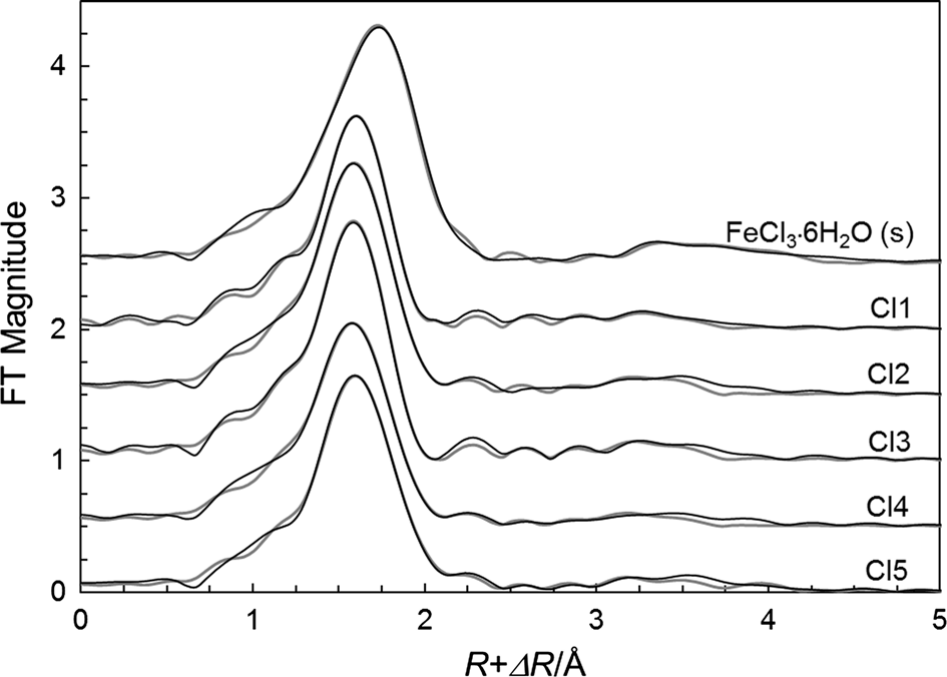
Fitting of the Fourier transforms, without correction for the phase shift, using the structure parameters summarized in Table 2

The hydrated iron(III) ion binds six water molecules in a regular octahedron with a mean Fe–O bond distance of 1.993 Å in the solid state (Table S2) as well as in aqueous solution [34]. The structure of the octahedral hexachloroferrate complex, [FeCl_6_]^3−^, has been reported in a number of solids with a mean Fe–Cl bond distance of 2.393 Å, Table S2. The Fe–Cl and Fe–O bond distances in the studied aqueous solutions, being fairly close to those in the octahedral [Fe(H_2_O)_6_)]^3+^ and [FeCl_6_]^3−^ complexes (Table 2) strongly indicate octahedral configuration of the studied ferric chloride complexes. The EXAFS data of solid *trans*-[FeCl_2_(H_2_O)_4_]Cl·2H_2_O show slightly shorter Fe–Cl and Fe–O bond distances than reported in the crystallographic study [23] (Table 2). The mean Fe–Cl bond distance in [FeCl_2_(H_2_O)_4_]Cl·2H_2_O, 2.278 Å (this study)/2.292 Å [23], is much shorter than in the [FeCl_6_]^3−^ complex, while the mean Fe–O bond distance, 2.057 Å/2.070 Å is significantly longer than in the hydrated iron(III) ion 1.99 Å (Table S2) showing that chloride ions are more strongly bound than the water molecules. The fit of the experimental data and Fourier transforms are given Figs. 1 and 2, respectively.

**Table 2:** Mean bond distances, *d*/Å, Debye–Waller factors, *σ*^2^, number of distances, *N*, the threshold energy, *E*_o_/eV, and the amplitude reduction factor, $S_{\text{o}}^{2}$, of the studied aqueous solutions of iron(III) chloride with varying concentration as determined by EXAFS in the *k* range 2–14 Å^−1^ at ambient room temperature

Solvent interaction	*N*	*d*	*σ* ^2^	*E*_o_	$S_{\text{o}}^{2}$
[FeCl_2_(OH_2_)_4_]Cl·2H_2_O(s)					
Fe–O	4	2.057(3)	0.0043(4)	7123.7(3)	0.78(2)
Fe–Cl	2	2.278(2)	0.0032(3)		
Square planar FeO_4_ MS	3 × 4	4.118(14)	0.0037(2)		
*trans*-FeCl_2_ MS	2 × 2	4.584(12)	0.0041(14)		
Solution Cl1: 1.00 mol·dm^−3^ Fe^3+^ + 1.50 mol·dm^−3^ Cl^−^ + 1.60 mol·dm^−3^ ${\text{ClO}}_{4}^{ - }$ in aqueous solution					
Fe–O	4.9(2)	2.007(2)	0.0028(3)	7123.7(3)	0.75(2)
Fe–Cl	1.1(2)	2.236(4)	0.0040(6)		
Fe–O–O MS	20	3.55(4)	0.007(3)		
Square planar FeO_4_ MS	3 × 4	4.009(16)	0.005(2)		
Solution Cl2: 1.00 mol·dm^−3^ Fe^3+^ + 3.00 mol·dm^−3^ Cl^−^ in aqueous solution, no acid added					
Fe–O	4.4	2.014(2)	0.0044(2)	7122.1(3)	0.72(2)
Fe–Cl	1.6	2.251(2)	0.0039(3)		
Square planar FeO_4_ MS	3 × 4	4.01(2)	0.012(3)		
*trans*-FeCl_2_ MS	2 × 0.9	4.52(3)	0.009(4)		
Solution Cl3: 1.00 mol·dm^−3^ Fe^3+^ + 3.00 mol·dm^−3^ Cl^−^ + 1.00 mol·dm^−3^ ${\text{ClO}}_{4}^{ - }$ in aqueous solution					
Fe–O	4.4	2.002(2)	0.0043(3)	7122.0(3)	0.81(2)
Fe–Cl	1.6	2.245(3)	0.0075(5)		
Square planar FeO_4_ MS	3 × 4	4.02(2)	0.009(3)		
*trans*-FeCl_2_ MS	2 × 0.9	4.54(3)	0.014(4)		
Solution Cl4: saturated solution (2.33 mol·dm^−3^) of FeCl_3_ in aqueous perchloric acid					
Fe–O	4.2	2.017(3)	0.0042(4)	7122.6(3)	0.71(2)
Fe–Cl	1.8	2.257(3)	0.0058(4)		
Square planar FeO_4_ MS	3 × 4	4.01(2)	0.009(4)		
*trans*-FeCl_2_ MS	2 × 1.4	4.56(2)	0.011(4)		
Solution Cl5: 1.00 mol·dm^−3^ Fe^3+^ + 4.00 mol·dm^−3^ Cl^−^ in aqueous solution, no acid added					
Fe–O	4.3	2.019(2)	0.0026(2)	7121.7(3)	0.67(2)
Fe–Cl	1.7	2.251(2)	0.0054(3)		
Square planar FeO_4_ MS	3 × 4	3.99(2)	0.009(3)		
*trans*-FeCl_2_ MS	2 × 1.7	4.56(3)	0.011(4)

**Fig. 3:**
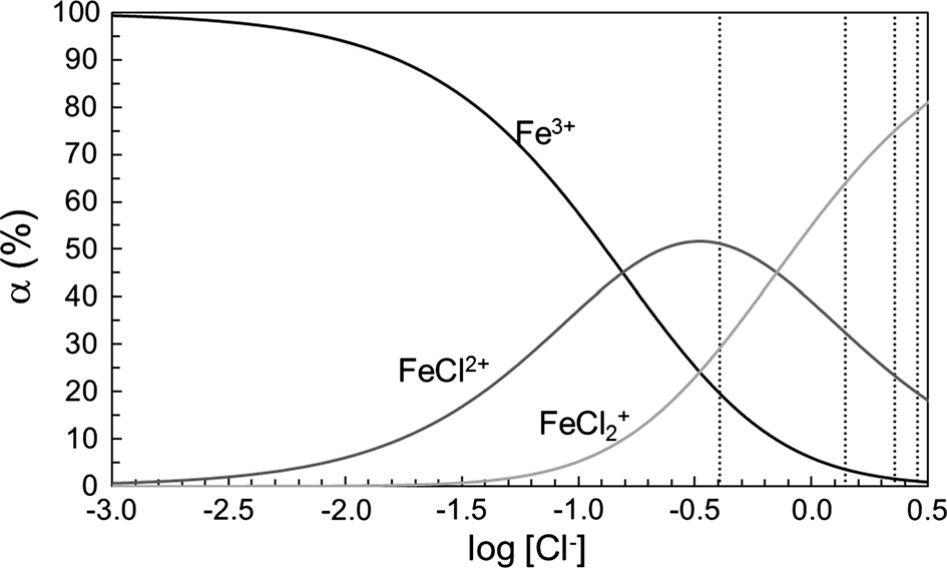
Complex distribution of iron(III) chloride in aqueous solution, based on the data reported in Ref. [31]. Black line, hydrated iron(III) ion; dark grey line, hydrated FeCl^2+^ complex; and light grey line, hydrated ${\text{FeCl}}_{2}^{ + }$ complex. The vertical dotted lines represent the solutions studied: solution Cl1 (log_10_ [Cl^−^] = − 0.394), solutions Cl2 and Cl3 (log_10_ [Cl^−^] = 0.145), solution Cl4 (log_10_ [Cl^−^] = 0.355) and solution Cl5 (log_10_ [Cl^−^] = 0.453)

**Fig. 4:**
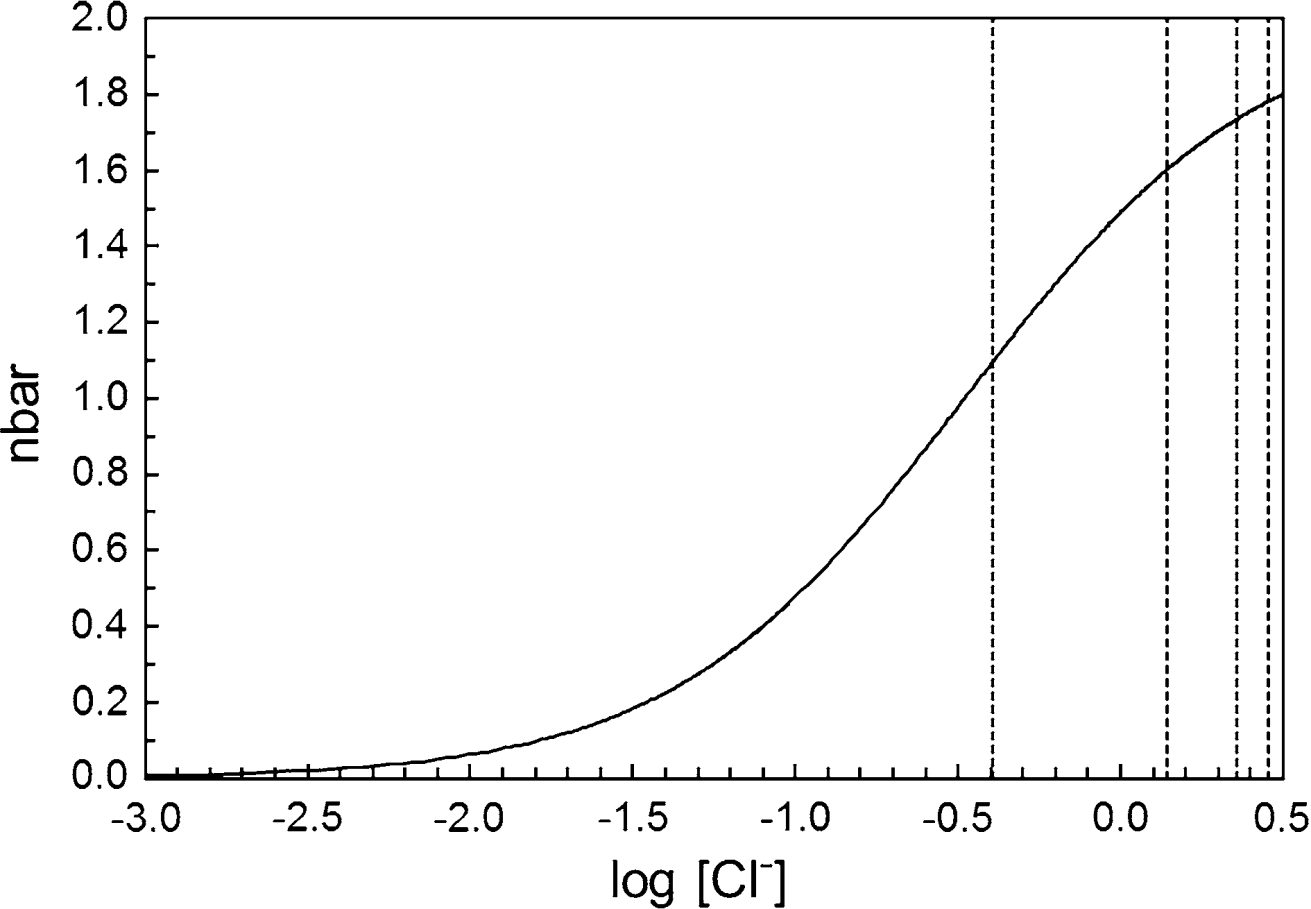
Complex formation function $\bar{n}$ of iron(III) chloride in aqueous solution, based on the data reported in Ref. [31]. The vertical dotted lines represent the solutions studied: Cl1 (log_10_ [Cl^−^] = −0.394), Cl2 and Cl3 (log_10_ [Cl^−^] = 0.145), Cl4 (log_10_ [Cl^−^] = 0.355) and Cl5 (log_10_ [Cl^−^] = 0.453)

The estimated composition/complex distribution of the aqueous iron(III)-chloride solutions in this study is shown in Fig. 3, and the complex formation function in Fig. 4, using the stability constants reported by Strahm et al. [35]. The Cl1 solution has an approximate ligand number, the mean number of chloride ions bound per iron(III) ion denoted by $\bar{n}$, of 1.10, and contains ca. 20% [Fe(H_2_O)_6_]^3+^, 51% [FeCl(H_2_O)_5_]^2+^ and 29% [FeCl_2_(H_2_O)_4_]^+^ complexes. The mean Fe–O bond distance, 2.007(4) Å, and the corresponding multiple scattering distance (Table 1) show that all complexes in solution Cl1 are octahedral, and that the Fe–O bond distance is slightly longer than in the hydrated iron(III) ion. The mean Fe–Cl bond distance, 2.236(8) Å, is significantly shorter than in *trans*-[FeCl_2_(H_2_O)_4_]Cl·2H_2_O [23] and slightly shorter than in *cis*-[FeCl_2_(H_2_O)_4_][FeCl_4_]·H_2_O [25]. As the dominating species in the Cl1 solution is the [FeCl(H_2_O)_5_]^2+^ complex (Fig. 3) it can be concluded that the structure of the [FeCl(H_2_O)_5_]^2+^ complex is octahedral, and the Fe–Cl and Fe–O bond distances are slightly shorter and longer, respectively, than in the *trans*-[FeCl_2_(H_2_O)_4_]^+^ complex in aqueous solution and in solid *trans*-[FeCl_2_(H_2_O)_4_]Cl·2H_2_O.

The Cl2 and Cl3 solutions have an approximate ligand number of 1.60 (Fig. 4) containing ca. 10% [Fe(H_2_O)_6_]^3+^, 45% [FeCl(H_2_O)_5_]^2+^ and 45% [FeCl_2_(H_2_O)_4_]^+^ complexes (Fig. 3). The mean Fe–Cl and Fe–O bond distances, 2.24(1) and 2.01(1) Å, respectively, and the corresponding multiple scattering at double bond distance, Table 2, show that the hydrated FeCl^2+^ and ${\text{FeCl}}_{2}^{ + }$ complexes in solutions Cl2 and Cl3 are octahedral. The strong linear Fe–Cl–Cl and Fe–Cl–Fe–Cl multiple scattering shows that the [FeCl_2_(H_2_O)_4_]^+^ complex is mainly present as the *trans* complex. The obtained Fe–Cl bond distance in solutions Cl2 and Cl3 show that the Fe–Cl bond is slightly longer in [FeCl_2_(H_2_O)_4_]^+^ than in [FeCl(H_2_O)_5_]^2+^ and that the structure of the [FeCl_2_(H_2_O)_4_]^+^ complex in aqueous solution is in good agreement with the structure parameters observed in solid *trans*-[FeCl_2_(H_2_O)_4_]Cl·2H_2_O [23].

The Cl4 and Cl5 solutions have approximate ligand numbers of 1.78 and 1.73, respectively, Fig. 4, with [FeCl_2_(H_2_O)_4_]^+^ as the dominating species, 79 and 75%, respectively. The obtained mean Fe–Cl and Fe–O bond distances confirm the structure of the [FeCl_2_(H_2_O)_4_]^+^ complex in solutions Cl2 and Cl3, discussed above, and that higher complexes than [FeCl_2_(H_2_O)_4_]^+^ are not formed in any significant amounts at very high concentrations, solution Cl4, or at large excess of chloride, 1.0 mol·dm^−3^, solution Cl5.

## Conclusions

This study shows clearly that higher chloroferrate(III) complexes are not formed in aqueous solution, independent of total ferric chloride concentration or excess concentration of chloride ions at room temperature. The dominant species in concentrated aqueous solution of ferric chloride and in solutions with an excess of chloride ions, less than 1 mol·dm^−3^, is the *trans*-[FeCl_2_(H_2_O)_4_]^+^ complex. Previous complex formation studies (Table S1) show that the dominating species in dilute aqueous solution of ferric chloride, less than 1 mol·dm^−3^, are the hydrated iron(III) and chloride ions (Figs. 3 and 4).

## Electronic supplementary material

Below is the link to the electronic supplementary material.

Supplementary material 1 (DOCX 50 kb)
