# Supplementary material for: Ferric Chloride Complexes in Aqueous Solution: An EXAFS Study
Source: J Solution Chem. 2018 May 5;47(5):797–805. doi: 10.1007/s10953-018-0756-6 (PMC5973949; doi:10.1007/s10953-018-0756-6)
Supplement: Supplementary file 1 — Supplementary material 1 (DOCX 50 kb) [file 10953_2018_756_MOESM1_ESM.docx]

Supplementary Material for

# Ferric Chloride Complexes in Aqueous Solution: A Structural Study

**Ingmar Persson**

Department of Molecular Sciences, Swedish University of Agricultural Sciences, P.O.Box 7015, 750 07 Uppsala, Sweden

**Table S1** Summary of selected complex formation studies on the iron(III)–chloride system in water, *N*-methylacetamide, *N,N*-dimethylformamide and dimethylsulfoxide

log_10_ *K*_1_ log_10_ *K*_2_ log_10_ *K*_3_ Method Ionic medium ^a^ Reference

Water

0.48 emf Variable 1

0.61 emf None 2

0.62 0.11 –1.4 spectroph. 1.0 M NaClO_4_ 3

0.76 0.30 –0.06 spectroph. 2.0 M NaClO_4_ 4

0.61 0.18 spectroph. 1.2 M NaClO_4_ 5

0.62 spectroph. 2.5 M NaClO_4_ 6

–0.7 –1.4 anion exch. 1-12 M LiCl 7

0.78 distribution 0.2 M NaClO_4_ 8

0.64 spectroph. 0.5 M NaClO_4_ 9

0.47 spectroph. 1.0 M NaClO_4_ 10

0.63 0.11 distribution 1.0 M HClO_4_ 11

0.66 0.15 0.02 spectroph. 1.2 M NaClO_4_ 12

0.6 spectroph. 0.15 M NaClO_4_ 13

0.75 spectroph. 0.5 M NaClO_4_ 14

0.7 nmr/esr Variable 15

0.88 -0.08 distribution 4.0 M NaClO_4_ 16

0.48 0.11 kinetic 1.0 M 17

0.47 spectroph. 1.0 M HClO_4_ 18

0.81 0.26 spectroph. 2.6 M NaClO_4_ 19

0.49 spectroph. 0.4 M NaClO_4_ 20

0.74 spectroph. 1.0 M NaClO_4_ 21

0.58 1.0 M HClO_4_ 22

0.67 0.70 spectroph. 0.1 M NaClO_4_ 23

1.42 0.66 –1.26 spectroph. 24

1.52 1.87 0.77 spectroph. 25

- - - - - - - - - - - - - - - - - - - - - - - - - - - - - - - - - - - - - - - - - - - - - - - - - - - - - - - - - - - - - - - - - - - -

log_10_ *K*_1_ log_10_ *K*_2_ Method Ionic medium ^a^ Reference

N-Methylacetamide

2.88 1.11 spectroph. 26

- - - - - - - - - - - - - - - - - - - - - - - - - - - - - - - - - - - - - - - - - - - - - - - - - - - - - - - - - - - - - - - - - - - -

N,N-Dimethylformamide

4.30 3 Spectroph. 0.2 M NaClO_4_ 27

- - - - - - - - - - - - - - - - - - - - - - - - - - - - - - - - - - - - - - - - - - - - - - - - - - - - - - - - - - - - - - - - - - - -

Dimethylsulfoxide

3.72 2.04 spectroph. 0.1 M NaClO_4_ 28

3.62 2.10 spectroph. 0.1 M NaClO_4_ 29

4.74 2.45 spectroph. 0.1 M NaClO_4_ 30

^a^ ‘M’ denoted the concentration in mol·dm^–3^

# Table S2 Summary of Fe-O bond distances in hydrated iron(III) ions in reported solid state structures. In the first column the ICSD (digits), ref. 31, or CSD code (letters), ref. 32, aee given

### 1002 1.986 Å [Fe(H_2_O)_6_](NO_3_)_3_.3H_2_O

68910 1.989 Å Cs[Fe(H_2_O)_6_](SeO_4_)_2_.6H_2_O

262788 1.990 Å [Fe(H_2_O)_6_](NO_3_)_3_

420959 1.990 Å [Fe(H_2_O)_6_](B(CN)_4_)_3_

XIGSEL 1.991 Å [Fe(H_2_O)_6_]_2_[Fe_2_H_20_O_11_](NO_3_)_10_.4C_10_H_20_O_5_.6H_2_O

69132 1.991 Å Na[Fe(H_2_O)_6_](SO_4_)_2_

81103 1.992 Å Cs[Fe(H_2_O)_6_][Fe_3_(H_2_O)_6_(SeO_4_)_6_].6H_2_O

69435 1.994 Å Cs[Fe(H_2_O)_6_](SO_4_)_2_.6H_2_O

201213 1.995 Å Cs[Fe(H_2_O)_6_](SO_4_)_2_.6H_2_O

MEWZAP 1.996 Å [Fe(H_2_O)_6_](C_9_H_9_O_9_S_3_)_3_.3(CH_3_OH)

MEWZAP01 1.996 Å [Fe(H_2_O)_6_](C_9_H_9_O_9_S_3_)_3_.3(CH_3_OH)

69435 2.001 Å Cs[Fe(H_2_O)_6_](SO_4_)_2_.6H_2_O

237717 2.007 Å [Fe(H_2_O)_6_](ClO_4_)_3_.3H_2_O

**Mean 1.993 Å/12 structures**

# Table S3 Summary of Fe–Cl and Fe–O bond distances in hydrated chloroiron(III) complexes in reported solid state structures. In the first column the ICSD (digits) or CSD code (letters) are given

# [FeCl(solv)_5_] complexes

161590 2.278 + 2.042 Å [FeCl(OS(CH_3_)_2_)_5_][Cl_3_FeOFeCl_3_]

161590 2.291 + 2.028 Å [FeCl(OS(CH_3_)_2_)_5_][Cl_3_FeOFeCl_3_]

**Mean 2.285 + 2.035 Å/2 structures**

# *cis*-[FeCl_2_(solv)_4_]^+^ complexes

8198 2.244 + 2.063 Å [FeCl_2_(OH_2_)_4_][FeCl_4_]∙H_2_O

DIJSAR 2.273 + 2.051 Å [FeCl_2_(OH_2_)_4_][OsCl_3_(CO)_3_]·2H_2_O

WEKYIV 2.277 + 2.051 Å [FeCl_2_(OS(CH_3_)_2_)_4_][Ru(CO)_3_Cl_3_]∙2H_2_O

DIJSAR 2.277 + 2.051 Å [FeCl_2_(OH_2_)_4_][RuCl_3_(CO)_3_]·2H_2_O

SINZAS 2.309 + 2.072 Å [Fe_3_C_36_H_36_N_6_O_16_][FeCl_6_(H_2_O)_2_]Br_6_·12H_2_O

**Mean 2.276 + 2.035 Å/4 structures**

# *trans*-[FeCl_2_(solv)_4_]^+^ complexes

JADZIX 2.350 + 2.012 Å [FeCl_2_(OP(C_6_H_5_)_3_)_4_][FeCl_4_]

24551 2.364 + 2.077 Å [FeCl_2_(OH_2_)_4_][SbCl_6_]∙4H_2_O

FEDMSI10 2.366 + 2.006 Å [FeCl_2_(OS(CH_3_)_2_)_4_][FeCl_4_]

**Mean 2.360 + 2.032 Å/3 structures**

# *mer*-[FeCl_3_(solv)_3_] complexes

GOPSIN 2.330 + 2.047 Å (C_6_H_14_N_2_)[*mer-*FeCl_3_(OH_2_)_3_]Cl_2_

YITVON 2.331 + 2.060 Å (C_4_H_12_N_2_)[*mer-*FeCl_3_(OH_2_)_3_]Cl_2_

GOPSIN01 2.336 + 2.051 Å (C_6_H_14_N_2_)[*mer-*FeCl_3_(OH_2_)_3_]Cl_2_

**Mean 2.332 + 2.053 Å/3 structures**

# *fac*-[FeCl_3_(solv)_3_] complexes

KAPLIX 2.315 + 2.069 Å (C_18_H_15_N_6_)[*fac-*FeCl_3_(OH_2_)_3_]Cl_3_∙3H_2_O

# [FeCl_3_(solv)_2_] complexes

KATJOE 2.201 + 2.109 Å [FeCl_3_(OC_4_H_8_)_2_]

UJIMUV 2.204 + 2.144 Å [FeCl_3_(O(C_2_H_5_)_2_]

**Mean 2.203 + 2.077 Å/2 structures**

# [FeCl_4_(solv)_2_]^–^ complexes

GOPSOT 2.358 + 2.059 Å (C_6_H_14_N_2_)_2_[FeCl_4_(OH_2_)_2_][FeCl_4_]Cl_2_

VOBYEQ 2.365 + 2.049 Å (C_6_H_14_N_2_)_2_[FeCl_4_(OH_2_)_2_]Cl_3_

**Mean 2.362 + 2.054 Å/2 structures**

# [FeCl_5_(H_2_O)]^2-^ complex

VOCBAQ 2.358 + 2.180 Å (H_3_NCH_2_CH_2_NH_3_)[FeCl_5_(H_2_O)]

VOCBAQ01 2.360 + 2.170 Å (H_3_NCH_2_CH_2_NH_3_)[FeCl_5_(H_2_O)]

23318 2.361 + 2.176 Å Cs_2_[FeCl_5_(H_2_O)]

65591 2.362 + 2.137 Å Cs_3_[FeCl_5_(H_2_O)]Cl

81017 2.362 + 1.943 Å K_2_[FeCl_5_(H_2_O)]

81021 2.362 + 2.043 Å K_2_[FeCl_5_(D_2_O)]

81022 2.364 + 2.146 Å Rb_2_[FeCl_5_(D_2_O)]

200322 2.366 + 2.100 Å (NH_4_)_2_[FeCl_5_(H_2_O)]

GIFJUB 2.367 + 2.146 Å (C4H_16_N_3_)_2_[FeCl_5_(H_2_O)]Cl

31873 2.368 + 2.107 Å (H_3_O)_2_[FeCl_5_(H_2_O)]

HAGMIL01 2.369 + 2.094 Å (C_10_H_28_N_4_)[FeCl_5_(H_2_O)]Cl_2_·H_2_O

HAGMEH 2.370 + 2.147 Å (C_10_H_28_N_4_)[FeCl_5_(H_2_O)]Cl_2_

79782 2.372 + 2.070 Å K_2_[FeCl_5_(H_2_O)]

**Mean 2.365 + 2.112 Å/13 structures**

# [FeCl_4_]^–^ complex

TOQDAE 2.161 Å (H_3_N(CH_2_)_6_NH_3_)_4_[FeCl_4_]_2_[FeCl_6_]Cl_4_

171652 2.176 Å N(SO_2_N(CH_3_)_3_)_2_[FeCl_4_]

159487 2.178 Å (CH_3_)_4_N[FeCl_4_]

22232 2.185 Å PCl_4_[FeCl_4_]

240608 2.186 Å (CH_3_)_2_NH_2_)_2_[FeCl_4_]Cl

HITVOV 2.187 Å (C_6_H_16_N_2_)_6_[FeCl_4_]_2_[FeCl_4_]_2_[FeCl_5_][FeCl_6_]

1267 2.189 Å Cs[FeCl_4_]

33534 2.189 Å NO[FeCl_4_]

62913 2.189 Å H[FeCl_4_]·6H_2_O

63120 2.189 Å (H_3_O(H_2_O)_5_)[FeCl_4_]

KAPLET 2.189 Å [FeCl_4_]_3_[FeCl_6_]

6382 2.190 Å [Fe(NCD)_6_][FeCl_4_]

182 2.191 Å [Fe(NCD)_6_][FeCl_4_]

8198 2.193 Å [FeCl_2_(OH_2_)_4_][FeCl_4_]∙H_2_O

26401 2.194 Å S_6_N_4_[FeCl_4_]_2_

47103 2.194 Å S_4_N_4_Cl[FeCl_4_]

62685 2.194 Å (H_9_O_4_)[FeCl_4_]

172864 2.195 Å (ClTeN_2_S_2_)[FeCl_4_]

16994 2.196 Å Na[FeCl_4_]

75334 2.197 Å ((SeCl)_2_N)[FeCl_4_]

**Mean 2.188 Å/20 structures**

# [FeCl_6_]^3–^complex

DALLIL 2.385 Å (CH_5_N_2_)_3_[FeCl_6_]

PIMXIS 2.388 Å [CoC_6_H_24_N_6_]_3_[FeCl_6_]Cl_6_·H_2_O

PIMXIS01 2.389 Å [CoC_6_H_24_N_6_]_3_[FeCl_6_]Cl_6_·H_2_O

KAPLET 2.391 Å (C_18_H_15_N_6_)_2_[FeCl_4_]_3_[FeCl_6_]

TOQCUX 2.391 Å (CH_3_NH_3_)_4_[FeCl_6_]Cl

32718 2.393 Å [Co(NH_3_)_6_][FeCl_6_]

TOQDAE 2.394 Å (H_3_N(CH_2_)_6_NH_3_)_4_[FeCl_4_]_2_[FeCl_6_]Cl_4_

HITVOV 2.395 Å (C_6_H_16_N_2_)_6_[FeCl_4_]_2_[FeCl_4_]_2_[FeCl_5_][FeCl_6_]

KAPLOD 2.398 Å (C_18_H_15_N_6_)[FeCl_6_]

PIMXEO 2.400 Å [CrC_6_H_24_N_6_]_3_[FeCl_6_]Cl_6_·H_2_O

PIMXEO01 2.401 Å [CrC_6_H_24_N_6_]_3_[FeCl_6_]Cl_6_·H_2_O

**Mean 2.393 Å/11 structure**

**Refrences**

1. Popoff, S., Kunz, A. H.: [Oxidation-reduction potentials. I. The ferric–ferrous electrode](http://pubs.acs.org/doi/10.1021/ja01377a007). J. Am. Chem. Soc. **51**, 382–394 (1929)
2. Møller, M.: The [complex formation of ferric ions with chloride ions](http://pubs.acs.org/doi/10.1021/j150386a010). J. Phys. Chem. **41**, 1123–1128 (1937)
3. Rabinowitch, E., Stockmayer, W.H.: Association of ferric ions with hloride, bromide and hydroxyl ions (a spectroscopic study). J. Am. Chem. Soc. **64**, 335–347 (1942)
4. Olerup, H.: Beräkning av jämviktskonstanter ur ljusabsorptionsmätningar. Järn(III)kloridens komplexitet. Sv. Kem. Tidskr. **55**, 324–333 (1943)
5. Lister, M.W., Rivington, D.E.: Some ferric halide complexes, and ternary complexes with thiocyanate ions. Can. J. Chem. **33**, 1603–1613 (1955)
6. Coll, H., Nauman, R.V., West, P.W.: [The stability of FeCl^++^ in perchlorate solutions](http://pubs.acs.org/doi/10.1021/ja01515a005). J. Am. Chem. Soc. **81**, 1284–1288 (1959)
7. Marcus, Y.: [The anion exchange of metal complexes—IV: The iron(III)–chloride system](https://www.sciencedirect.com/science/article/pii/0022190260803752). J. Inorg. Nucl. Chem. **12**, 287–296 (1960)
8. White, J.M., Kelly, P., Li, N.C.: [Dinonyl naphthalene sulphonic acid and tri-*n*-octylamine as liquid ion-exchangers for the study of Fe(III) and In(III)–chloride complexes.](https://www.sciencedirect.com/science/article/pii/0022190261805083) J. Inorg. Nucl. Chem. **16**, 337–344 (1961)
9. Sutin, N., Rowley, J.K., R.W. Dodson, R.W.: [chloride complexes of iron(III) ions and the kinetics of the chloride-catalyzed exchange reaction between iron(II) and iron(III) in light and heavy water](http://pubs.acs.org/doi/10.1021/j100825a037). J. Phys. Chem. **65**, 1248–1252 (1961)
10. Woods, S.M.J.M., Gallagher, P.K., King, E.L.:[Thermodynamics of association of iron(III) ion and chloride Ion in aqueous solution](http://pubs.acs.org/doi/10.1021/ic50001a011). Inorg. Chem. **1**, 55–65 (1962)
11. D. F. C. Morris, D. F. C, Wilson, A. R.: Application of dinonyl naphthalene sulphonic acid in solution in *n*-heptane as a liquid cation-exchanger to a study of the formation of iron(III) chloride and iron(III) bromide complexes. J. Inorg. Nucl. Chem. **31**, 1532–1536 (1969)
12. Masłowska, J.: Mixed complexes of iron(III). Part IV. Equilibria in the system: Fe(ClO_4_)_3_–NaCl–Na_2_SO_4_–H_2_O. Rocz. Chem. **43**, 15–24 (1969)
13. Fordham, A.W.: [The formation of hydroxy and chloro complexes of iron(III) in chloride and perchlorate media](http://www.publish.csiro.au/CH/CH9691111). Aust. J. Chem. **22**, 1111–1122 (1969)
14. Rowley, J.K., Sutin, N.: [Formation and dissociation of monochloroiron(III) at high ionic strengths: equilibrium and kinetic measurements](http://pubs.acs.org/doi/10.1021/j100909a001). J. Phys. Chem. **74**, 2043–2054 (1970)
15. Levanon, H., Stein, G., Luz, Z.: ESR study of complex formation and electronic relaxation of Fe^3+^ in aqueous solutions. J. Chem. Phys. **53**, 876–886 (1970)
16. Sekine, T., Tetsuka, T.: [The solvent extraction of iron(III) in perchlorate solutions containing chloride or bromide Ions with 2-ghenoyltrifluoroacetone and trioctylphosphine oxide](http://www.journal.csj.jp/doi/abs/10.1246/bcsj.45.1620). Bull. Chem. Soc. Jpn. **45**, 1620–1625 (1972)
17. Schwarz, H.A., Dodson, R.W.: [Kinetics of dissociation of ferric chloride complexes. Stability constants of inner- and outer-sphere complexes](http://pubs.acs.org/doi/10.1021/j100566a017). J. Phys Chem. **80**, 2801–2804 (1976)
18. Perlmutter-Hayman, B., Tapuhi, E.: Formation constants of other sphere complexes at high ionic strength. A re-evaluation of kinetics results. J. Coord. Chem. **8**, 75–79 (1978)
19. Strahm, U., Patel, R.C., Matijevic, E.: Thermodynamics and kinetics of aqueous iron(III) chloride complexes formation. J. Phys. Chem. **83**, 1689–1695 (1979)
20. Feng, Q., Waki, H.: [Evaluation of absolute stability constants of complexes - I. Theory and applications to the inorganic one-to-one complexes of iron(III) in perchlorate media](https://www.sciencedirect.com/science/article/pii/S0277538700804696). Polyhedron **7**, 291–295 (1988)
21. Brubaker, G.R., Peterson, R.A.: [Stability of the monochloroiron(III) cation in aqueous solutions between 298 and 398 K](https://www.sciencedirect.com/science/article/pii/S0020169300892932). Inorg. Chim. Acta **155**, 139–144 (1989)
22. Inada, Y., Funahashi, S.: EXAFS, complexation, formation constant, iron(III) ion, chloride ion. Z. Naturforsch., Teil B **54**, 1517–1523 (1999)
23. Byrne, R., Yao, W., Luo, Y., Wang, B.: [The dependence of Fe^III^ hydrolysis on ionic strength in NaCl solutions](https://www.sciencedirect.com/science/article/pii/S0304420305000940). Marine Chem. **97**, 34–48 (2005)
24. Stefánsson, A., Lemke, K. H., Seward, T. M.: International Conference on the Properties of Water and Steam, Berlin, September 8-11, 2008, Proceeedings;

Available at: <http://www.15icpws.de/papers/09_Geo-05_stefansson.pdf>

1. Tagirov, B.R., Diakonov, I.I., Devina, O.A., Zotov, A.V.: Standard ferric–ferrous potential and stability of FeCl^2+^ to 90 °C. Thermodynamic properties of Fe^3+^(aq) and ferric–chloride species. Chem. Geol. **162,** 193–129 (2000)
2. Reynolds, W.L., Weiss, R.: [Iron(III) complexes in non-aqueous solvents. I. The solvolysis and chloride complex constants in N-methylacetamide](http://pubs.acs.org/doi/10.1021/ja01517a005). J. Am. Chem. Soc. **81**, 1790–793 (1959)
3. Wada, G., Sahira, Y., Ohsaki, K., Shinoda, F.: [Kinetic studies of the electron transfer reaction in iron(II) and iron(III) systems. VI. Reaction in N,N-dimethylformamide](http://www.journal.csj.jp/doi/abs/10.1246/bcsj.47.851). Bull. Chem. Soc. Jpn. **47**, 851–854 (1974)
4. Wada, G., Reynolds, W.: [Isotope exchange in the iron(II)–iron(III)–chloride ion system in dimethyl sulfoxide](http://pubs.acs.org/doi/10.1021/ic50042a013). Inorg. Chem. **5**, 1354–1358 (1966)
5. Wada, G.: [Spectrophotometric determination of formation constants of iron(III) complexes with chloride ion in dimethyl sulfoxide](http://www.journal.csj.jp/doi/abs/10.1246/bcsj.41.882). Bull. Chem. Soc. Jpn. **41**, 882–888 (1968)
6. Linane, P.J., Hugus, Jr., Z.Z.: [Normal equations for the Gaussian least-squares refinement of formation constants with simultaneous adjustment of the spectra of the absorbing species](http://pubs.acs.org/doi/10.1021/ic50086a013). Inorg. Chem. **9**, 757–762 (1970)
7. Inorganic Crystal Structure Database, 1.4.6 (release: 2017-1); FIZ Karlsruhe/National Institute of Standards and Technology, U.S. Department of Commerce, and references therein
8. Allen, F.H.: [The Cambridge Structural Database: a quarter of a million crystal structures and rising](http://journals.iucr.org/b/issues/2002/03/01/an0610/index.html). Acta Crystallogr., Sect. B **58**, 380–388 (2002), and references therein
